# Aluminum for Dental Plates

**Published:** 1902-11

**Authors:** 


### Reviews of Dental Literature.

Aluminum for Dental Plates.—Ash & Sons' Quarterly Circular for June, 1902, contains a paper by A. Sandvig, of Lillehammer, Norway, on "Aluminum in Dental Practice." The author has for some years experimented with this metal for dental plates, and has devised a method of swaging aluminum upon Spence metal dies, using moulding-sand as a counter-die. He was led to abandon the usual zinc die and lead counter-die from observing that when the aluminum became contaminated with either metal, although the amount was so small as to escape observation, the plate was quickly destroyed by corrosion when placed in the mouth. Unless great care was used to keep the plate enveloped in tissue-paper during the swaging, and to carefully polish it before annealing, minute particles of zinc or lead adhered and united with the aluminum during annealing; these contaminated spots in a few months developed into holes. He has devised a swaging apparatus whereby the moulding-sand, confined within a cylinder, is forced down upon the aluminum plate in position on the Spence metal die by screw pressure. He states that the moulding-sand thus used becomes as effective a counter-die as is lead, and all risk of metallic contamination is avoided. He lays stress upon thoroughly swaging the aluminum so as to develop hardness, and states that, thoroughly hardened by swaging, the metal becomes elastic, hard, and eight times as strong. To this end he anneals as little as possible; if the metal is pure, not at all after beginning to form the plate. To strengthen the plate, he doubles it over the ridge by making a strip of metal conform to this part, perforating it, and with aluminum rivets riveting it in position with a strip of black rubber between it and the plate. He secures the teeth to the plate with vulcanite. He enjoins care to avoid the aluminum coming into contact with any other metal in the mouth except pure gold or platinum; these are harmless; any other metal excites a destructive galvanic corrosion. W. H. T.

